# Radiomic analysis of MRI to Predict Sustained Complete Response after Radiofrequency Ablation in Patients with Hepatocellular Carcinoma - A Pilot Study

**DOI:** 10.6061/clinics/2021/e2888

**Published:** 2021-07-08

**Authors:** Natally Horvat, Jose de Arimateia B. Araujo-Filho, Antonildes N. Assuncao-Jr, Felipe Augusto de M. Machado, John A. Sims, Camila Carlos Tavares Rocha, Brunna Clemente Oliveira, Joao Vicente Horvat, Claudia Maccali, Anna Luísa Boschiroli Lamanna Puga, Aline Lopes Chagas, Marcos Roberto Menezes, Giovanni Guido Cerri

**Affiliations:** I Departamento de Radiologia, Hospital Sirio Libanes, Sao Paulo, SP, BR; II Departamento de Radiologia, Instituto de Radiologia (InRad), Hospital das Clinicas (HCFMUSP), Faculdade de Medicina, Universidade de Sao Paulo, Sao Paulo, SP, BR; III Department of Radiology, Memorial Sloan Kettering Cancer Center, New York, USA; IV Instituto de Educacao e Pesquisa, Hospital Sirio Libanes, Sao Paulo, SP, BR; V Escola Politecnica, Universidade de Sao Paulo, Sao Paulo, SP, BR; VI Departamento de Engenharia Biomedica, Centro de Engenharia, Modelagem e Ciencias Sociais Aplicadas, Universidade Federal do ABC (UFABC), Santo Andre, SP, BR; VII Departamento de Gastroenterologia, Divisao de Gastroenterologia e Hepatologia Clinica, Hospital das Clinicas (HCFMUSP), Faculdade de Medicina, Universidade de Sao Paulo, Sao Paulo, SP, BR

**Keywords:** Carcinoma Hepatocellular, Magnetic Resonance Imaging, Radiomics, Radiofrequency Ablation

## Abstract

**OBJECTIVES:**

To investigate whether quantitative textural features, extracted from pretreatment MRI, can predict sustained complete response to radiofrequency ablation (RFA) in patients with hepatocellular carcinoma (HCC).

**METHODS:**

In this IRB-approved study, patients were selected from a maintained six-year database of consecutive patients who underwent both pretreatment MRI imaging with a probable or definitive imaging diagnosis of HCC (LI-RADS 4 or 5) and loco-regional treatment with RFA. An experienced radiologist manually segmented the hepatic nodules in MRI arterial and equilibrium phases to obtain the volume of interest (VOI) for extraction of 107 quantitative textural features, including shape and first- and second-order features. Statistical analysis was performed to evaluate associations between textural features and complete response.

**RESULTS:**

The study consisted of 34 patients with 51 treated hepatic nodules. Sustained complete response was achieved by 6 patients (4 with single nodule and 2 with multiple nodules). Of the 107 features from the arterial and equilibrium phases, 20 (18%) and 25 (23%) achieved AUC >0.7, respectively. The three best performing features were found in the equilibrium phase: Dependence Non-Uniformity Normalized and Dependence Variance (both GLDM class, with AUC of 0.78 and 0.76, respectively) and Maximum Probability (GLCM class, AUC of 0.76).

**CONCLUSIONS:**

This pilot study demonstrates that a radiomic analysis of pre-treatment MRI might be useful in identifying patients with HCC who are most likely to have a sustained complete response to RFA. Second-order features (GLDM and GLCM) extracted from equilibrium phase obtained highest discriminatory performance.

## INTRODUCTION

Liver cancer is the sixth most common cancer worldwide and the second leading cause of cancer mortality (1,2). Despite efforts in screening programs, early diagnosis of HCC remains challenging (3). Although surgical resection and transplant are the most effective curative treatment for HCC, they are not a feasible option for the majority of patients (4). In this context, several loco-regional therapies such as radiofrequency ablation (RFA) have received increasing attention as a therapeutic option for patients who are not eligible to undergo surgical resection or transplant, or as a bridging or downsizing modality before definitive treatment (5,6).

RFA consists of an alternating electric current that is applied to the nodule using an electrode needle. The electric current results in frictional heat leading to coagulative necrosis and irreversible cellular injury (7). RFA is indicated for patients with early-stage HCC and is considered a definitive treatment (8). However, some patients do not exhibit an immediate complete response to RFA or a sustained complete response during follow up. Furthermore, some patients present complications following RFA, including hepatic abscess ([Bibr B09] [Bibr B10] 9-11), biliary damage (12), hematoma, pseudoaneurysm, portal thrombosis, hepatic infarction and diaphragmatic hernia (10,11).

Radiomics is an emerging field in medical imaging that extracts quantitative data from conventional radiological imaging modalities and correlates them with several clinical outcomes, including treatment response ([Bibr B13] [Bibr B14] [Bibr B15] [Bibr B16] [Bibr B17] [Bibr B18] [Bibr B19] [Bibr B20] 13-21). Radiomics has the potential to detect specific characteristics of imaging that cannot be assessed visually. We hypothesize that MRI-based radiomic analysis of HCC may assist in predicting which patients will present sustained complete response after RFA. Awareness of the likely pattern of treatment response before RFA could improve the management of the patient with HCC by providing personalized treatment, and thus avoiding unnecessary procedures, reducing costs and optimizing available resources (22). Therefore, the purpose of our study was to investigate if quantitative textural features on contrast-enhanced MRI can predict treatment response after RFA in patients with HCC.

## MATERIALS AND METHODS

### Study population

**Figure 1:**
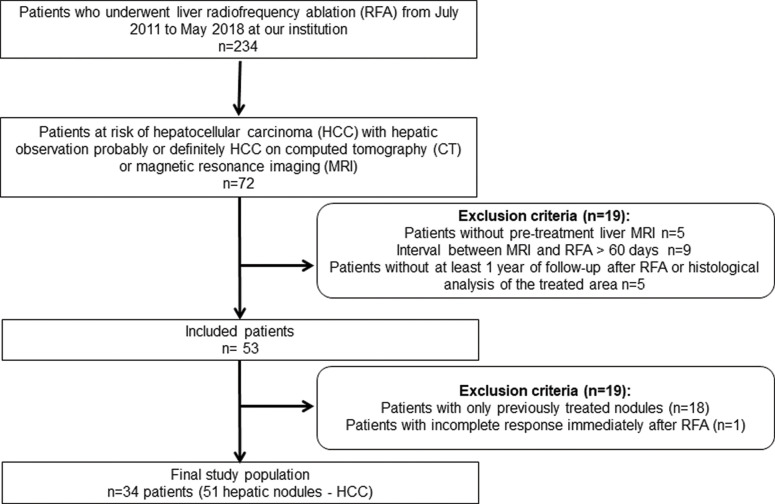
Flowchart summarizing the inclusion and exclusion criteria.

The institutional review board approved our retrospective study and waived the requirement for informed consent. Patients were selected from a maintained 6-year-database of consecutive patients who underwent both pretreatment MRI with a probable or definitive imaging diagnosis of HCC (LI-RADS 4 or 5) and loco-regional treatment with RFP. Exclusion criteria comprised: (a) interval between MRI and RFA longer than 60 days; (b) absence of posttreatment imaging follow up at least 1 year after RFA or histological evaluation of the treated area; (c) documented residual tumor immediately after RFA diagnosed in the first follow up image, acquired up to 15 days after RFA. The patient accrual is summarized in Figure 1.

### Declarations

Our institutional review board approved this retrospective study and waived the requirement for informed consent. The number of approval is 16618719.9.0000.0065.

### MR imaging protocol

The MR imaging examinations were performed at our institution. MR imaging scans were acquired on different GE Healthcare System platforms (GE Discovery MR750, GE Healthcare, Waukesha, WI and GE Signa HDxt, GE Healthcare, Waukesha, WI) using a phased-array coil. The minimum sequence required was axial post-contrast T1-weighted images (T1WI) at arterial and equilibrium phases without artifacts. MR imaging acquisition parameters used at our institution are summarized in Table 1.

### RFA protocol

All procedures were performed in an interventional suite with CT fluoroscopy and ultrasound capabilities under general endotracheal anesthesia assisted by an anesthesiologist. Radiofrequency (RF) ablation procedures used a Cool-tip™ RFA generator (Covidien, Mansfield, Massachusetts, USA) with single 17G RF electrode kits (ACT1530/ACT2030) or cluster RF electrode kits (ACCT1525). The RFA procedures were performed by an interventional radiologist with between 10 to 15 years’ experience.

### Clinical and laboratorial data

Clinical and laboratorial data were obtained from a detailed medical record review made by a radiologist with 3 years’ experience and a hepatologist with 10 years’ experience.

### Quantitative MR imaging texture analysis

#### Image segmentation

**Figure 2:**
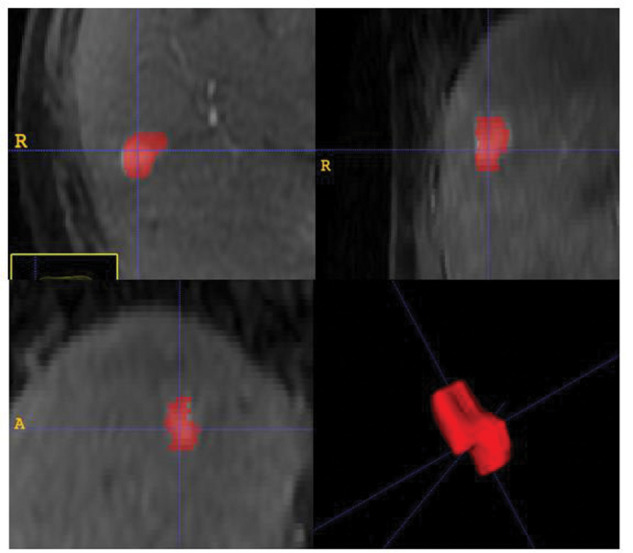
Multiplanar reconstruction illustrating the manual segmentation of the entire volume of interest of a liver lesion for extraction of quantitative features.

A radiologist, 6 years’ experience in liver MRI, manually segmented the hepatic nodules in all slices of T1 weighted pre-treatment MRI in both arterial and equilibrium phases. Quantitative textural features were extracted from the resulting volume of interest (VOI). Segmentation was performed using open-source software (ITK-SNAP, version 3.4.0; http://itksnap.org), as illustrated in Figure 2.

#### Textural feature extraction

A variety of textural features were extracted from the VOI and statistical analysis was used to identify those features which provide the best prediction of patient suitability for RAS. Our features can be classified as first-order features (FOF), shape-based features (SBF) and second-order features (SOF). FOF, such as mean or skewness, are calculated from the histogram of the VOI, and therefore do not take into account any spatial relationship between voxels. SBF are independent of gray level intensity, and describes the size and shape of the selected VOI. Second-order textural features, such as gray level co-occurrence matrix (GLCM), gray level size zone matrix (GLSZM), gray level run length matrix (GLRLM), neighboring gray tone difference matrix (NGTDM) and gray level dependence matrix (GLDM), take into account the spatial relationships between two or more voxels and can therefore be used to assess image texture (23,24).

A total of 107 textural features were calculated using the library Pyradiomics (https://www.radiomics.io/pyradiomics.html) and Python version 3.6 (https://www.python.org), consisting of: FOF, n=18; 3D shape-based, n=14; GLCM, n=24; GLRLM, n=16; GLSZM, n=16; NGTDM, n=5; and GLDM, n=14.

### Statistical analysis

Data are expressed as mean±standard deviation or median and interquartile range for continuous variables. Normality assumption was assessed graphically using Quantile-Quantile plots. Differences between patients with and without sustained complete response were assessed using Student's t-test or Wilcoxon test, as appropriate. Accounting for within-patient correlation, receiver operating characteristic (ROC) analysis of clustered nodules data was used to investigate the discriminatory power of each textural feature in identifying sustained complete response. To reduce the effect of influential values in the regression models, a Z-score transformation of textural features was applied. All statistical analyses were performed using R version 3.5.3 (R Foundation for Statistical Computing, Vienna, Austria) and a *p*-value<0.05 was considered statistically significant.

## RESULTS

### Clinical characteristics

The study consisted of 34 patients (mean age 67±9 years) with 51 treated hepatic nodules. Nearly half of the patients received a treatment for multiple nodules on the same day. The median interval between baseline MRI and RFA was 23 days. Patient characteristics are displayed in Table 2. The sustained complete response was achieved by 6 patients (4 with a single nodule and 2 with multiple nodes).

Regarding clinical data, patients with sustained complete response were predominantly female (83%, *p*=0.008), with better results of standardized prothrombin time [INR - international normalized ratio] (1.08±0.09 *vs* 1.19±0.13, *p*=0.031), albumin (4.2±0.2 *vs* 3.7±0.5, *p*=0.049), and total bilirubin [0.6 (0.4-0.9) *vs* 1.3 (0.7-2.4), *p*=0.025]. No other significant difference was found between groups with and without recurrence, including the etiology of liver disease and alpha-fetoprotein levels.

### MR textural analysis

**Figure 3:**
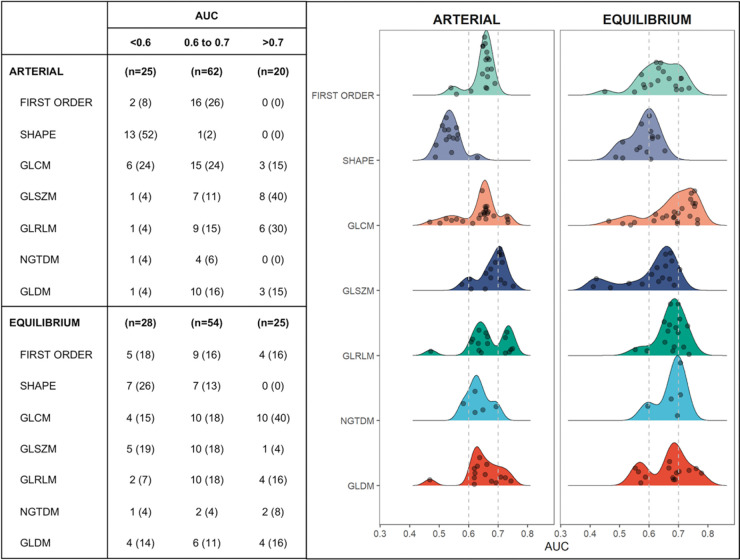
Discriminatory performance of textural features extracted from arterial and equilibrium phases of pre-treatment MRI grouped according to their area under the curve. GLCM (Gray Level Co-occurrence Matrix), GLSZM (Gray Level Size Zone Matrix), GLRLM (Gray Level Run Length Matrix), NGTDM (Neighboring Gray Tone Difference Matrix), and GLDM (Gray Level Dependence Matrix) are second order textural features.

**Figure 4:**
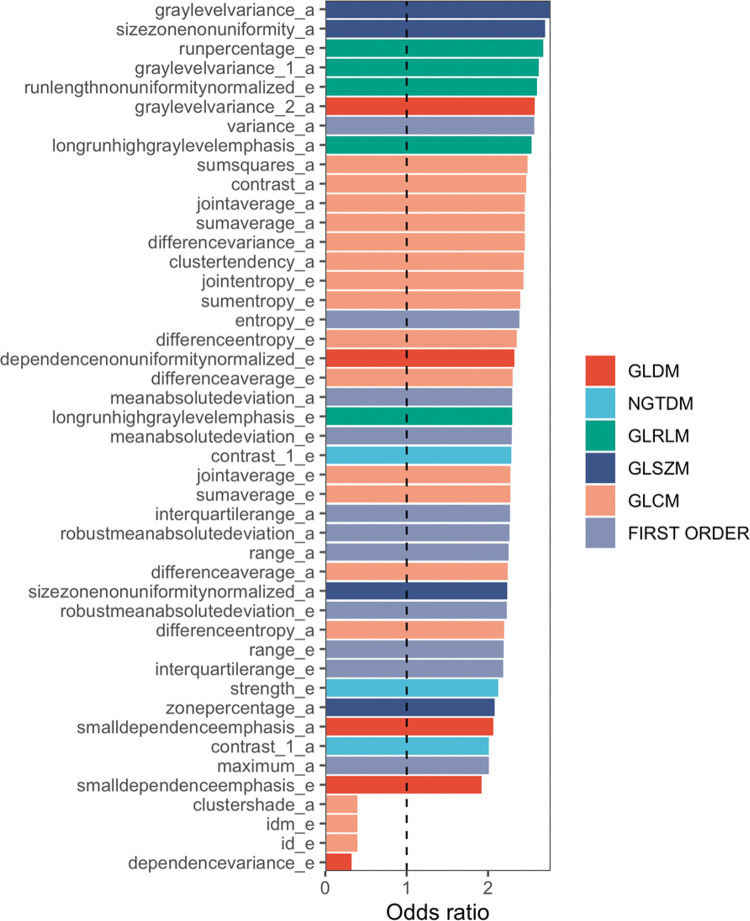
Features significantly (*p*<0.05) related to treatment response. When odds ratio >1, the higher their values the higher the chance for sustained response, whereas when odds ratio <1 the higher the values the lower the chance for sustained response. **a** (arterial phase), **e** (equilibrium phase). GLCM (Gray Level Co-occurrence Matrix), GLSZM (Gray Level Size Zone Matrix), GLRLM (Gray Level Run Length Matrix), NGTDM (Neighboring Gray Tone Difference Matrix), and GLDM (Gray Level Dependence Matrix) are second order textural features.

Figure 3 illustrates the discriminatory performance of textural features extracted from contrast phases grouped according to feature classes. Of the 107 features from the arterial and equilibrium phases, 20 (18%) and 25 (23%) provided area under the ROC curve (AUC) >0.7, respectively. Among those with best performance (AUC >0.7), only 4 (14%) features in the equilibrium phase were first order and none were shape features. GLSZM in the arterial phase and GLCM in the equilibrium phase were the classes with the most features that reached AUC >0.7. Individually, the 3 features with best discriminatory power were found in the equilibrium phase: Dependence Non-Uniformity Normalized and Dependence Variance (both GLDM class, with AUC of 0.78 and 0.76, respectively) and Maximum Probability (GLCM class, with AUC of 0.76). As can be seen in Figure 4, patients with sustained response were more likely to have higher values of Dependence Non-Uniformity Normalized compared to recurrent nodules.

## DISCUSSION

This is the first study to demonstrate that textural features, extracted from the entire volume of the nodule in pre-treatment MRI, can be used to determine the patients who are most likely to present sustained complete response to RFA. Overall, the second order features obtained from the equilibrium phase had higher discriminatory performance, with the best performers being the following: Dependence Non-Uniformity Normalized and Dependence Variance (both GLDM class, with AUC of 0.78 and 0.76, respectively) and Maximum Probability (GLCM class, with AUC of 0.76). None of the shape features extracted had AUC higher than 0.7. Only a few first order features reached AUC >0.7. Regarding clinical data, patients who presented sustained complete response were predominantly female (83%) and had more normal results of INR, albumin and total bilirubin when compared with patients who presented HCC recurrence.

Our results are in line with previous studies that evaluated radiomic features as a pre-treatment biomarker of treatment response of HCC after surgery and loco-regional treatment. Shan et al. evaluated the baseline CT of patients before ablation and hepatectomy (25), and demonstrated that the peritumoral radiomics features were able to predict treatment response with an AUC of 0.79. Other authors have also assessed radiomic features for predicting recurrence after hepatectomy ([Bibr B26] [Bibr B27] [Bibr B28] 26-29), transarterial chemoembolization (TACE) (30) and have obtained a similar performance. With regards to hepatectomy, two studies evaluated MRI in predicting recurrence and demonstrated an AUC that varied between 0.72 and 0.84 (26,29); while two other studies assessed CT-based radiomic features and obtained an AUC between 0.59 and 0.82 (27,28). Kim et al. evaluated both tumoral and peritumoral textural features and produced a clinicopathologic-radiomic model with a c-index of 0.716. Regarding loco-regional treatment, Park et al. (31) showed that HCC with response after TACE exhibited significantly different values of some textural features extracted from pretreatment CT images.

Our study demonstrated that SOF extracted from the nodule on pre-treatment MRI had higher discriminatory performance when compared with FOF and none of the SBF were significantly discriminatory. The biological interpretation of these results is challenging, particularly considering the preliminary nature of this study. Firstly, SBF, which reflect volume, size and geometry properties of the tumor had low discriminatory performance. This result runs against current medical practice since size is one of the most common criteria used to guide the treatment of HCC (32,33). Thus, our findings may reflect the complexity of HCC and suggest that size alone should not be used as the only criterion to determine the treatment of HCC.

Additionally, considering that FOF describe the distribution of the grayscale values, without taking into account the interaction of the pixels with their neighborhood, while SOF are determined by the relationship between the pixels and their neighborhood, the latter is able to provide better information regarding intra-tumoral heterogeneity (24). Indeed, tumor heterogeneity had a higher discriminatory performance in predicting sustained complete response, particularly the following features: Dependence Non-Uniformity Normalized, Dependence Variance and Maximum Probability. Again, counterintuitively, the current findings indicate that patients with sustained complete response had greater nodule heterogeneity (e.g. Dependence Non-Uniformity Normalized) compared to those with recurrence. Considering that in early hepatocarcinogenesis, HCC arises from a dysplastic nodule, and that smaller HCCs are selected to treat with RFA (33,34), we hypothesize that textural analysis may have demonstrated subtle heterogeneity patterns in nodules in their early phases of dedifferentiation, which may explain the higher sustained complete response among heterogeneous nodules. However, this conclusion is limited due to the retrospective nature of the study, the absence of histological correlation and the small sample size.

Currently, a multidisciplinary team is required to choose RFA based on rather limited information to predict suitability for RFA. Therefore, there may be a large clinical impact if the team was able to use radiomic feature information to guide their decision. Knowledge of the predicted response using radiomic features may influence the treatment decisions of the multidisciplinary team, such as whether to recommend RFA or whether to change the patients’ priorities on the liver transplant list, thereby allowing a more personalized treatment and improving the cost-effectiveness of HCC treatment.

The most important limitation in this study was the small sample size, in part due to the rigidity of our inclusion and exclusion criteria, which were set in order to achieve a more homogeneous group of patients. It was not possible to produce a statistical model to include the clinical data. Patients with sustained complete response were predominantly female and had more normal results of INR, albumin, and total bilirubin, which may be correlated with better liver function, and consequently improved treatment response or reduced tumorigenic potential. Larger studies are necessary to overcome these limitations and provide a better generalization of our results, possibly including clinical and laboratorial data in the prediction model and investigation of the independence of the statistical variables.

## CONCLUSION

This pilot study demonstrates that radiomics in pre-treatment MRI might be used to identify patients with HCC who are more likely to have a sustained complete response to RFA. The second-order features extracted from equilibrium phases obtained the highest discriminatory performance. Although encouraging, our results are preliminary and require validation on a larger dataset. After validation, the use of radiomic features may become an imaging biomarker to assist in the selection of patients to undergo RFA.

## AUTHOR CONTRIBUTIONS

Horvat N, Assuncao-Jr AN, Menezes MR, Cerri GG were responsible for the study conception and design. Horvat N, Araujo-Filho JAB, Rocha CCT and Oliveira BC were responsible for the literature search. Horvat N, Rocha CCT, Oliveira BC, Machado FAM, Horvat JV, Sims JA, Puga ALBL, Menezes MR and Araujo-Filho JAB were responsible for the image review. Rocha CCT, Oliveira BC, Maccali C and Chagas AL were responsible for the clinical information review. Assuncao-Jr An and Machado FAM were responsible for the statistical analysis. Horvat N, Araujo-Filho JAB, Sims JA, Chagas AL, Horvat JV, Maccali C and Menezes MR were responsible for the manuscript drafting and edition. Guido GG was responsible for the supervision. All of the authors approved the final version submitted of the manuscript.

## Figures and Tables

**Table 1:** Magnetic resonance imaging parameters.

	Seq	TR/TE (ms)	Matrix	FOV (mm)	ST/SG (mm)	BW (kHz)/ FA	Delay (s)
T1 arterial phase	GRE	3.4/1.7	320X160	360	5/1	90/12	20
T1 equilibrium phase	GRE	4.3/1.7	320X192	360	5/1	62/12	420

BW: bandwidth, TE: echo time, FA: flip angle, FOV: field of view, GRE: gradient echo, TR: repetition time, Seq: sequence, SG: section gap, ST: slice thickness T1WI: T1-weighted image.

**Table 2:** Patient characteristics.

	Total* (n=34)	Sustained Complete Response (n=6)	Recurrence (n=28)	*p*-value
Age [years]	67±9	67±9	67±9	0.570
Female, n (%)	11 (32)	5 (83)	6 (21)	0.008
BMI [kg/m^2^]	29±5	27±4	29±6	0.371
Symptoms, n (%)				
Splenomegaly	26 (76)	3 (50)	23 (82)	0.126
Ascites	5 (15)	1 (16)	5 (15)	0.998
Varices	24 (71)	4 (67)	20 (71)	0.997
Child-Pugh, n (%)				
A	30 (91)	6 (100)	24 (89)	0.999
B	2 (0)	0 (0)	2 (6)	0.999
C	1 (0)	0 (0)	1 (3)	0.999
MELD score	10±2	8±2	10±2	0.080
Multiple nodules	14 (41)	2 (33)	12 (43)	0.176
Etiology, n (%)				
HCV related	23 (67)	4 (67)	19 (67)	0.999
NASH related	4 (11)	1 (17)	3 (10)	0.558
Alcohol related	4 (11)	0 (0)	4 (14)	0.998
Others	3 (9)	1 (16)	2 (7)	0.452
Platelet count [thou/mm^3^]	99 (68-147)	102 (61-107)	106 (99-146)	0.838
INR*	1.17±0.13	1.08±0.09	1.19±0.13	0.031
Albumin (g/dL)	3.7±0.4	4.2±0.2	3.7±0.5	0.049
Total bilirubin (mg/dL)	1.1 (0.6-2.2)	0.6 (0.4-0.9)	1.3 (0.7-2.4)	0.025
Alpha-fetoprotein (ng/mL)	26 (5-101)	20 (3-53)	26 (6-122)	0.378

BMI: body mass index; MELD: model for end-stage liver disease.

* Plus-minus values are means±SD.

## References

[1] American Cancer Society (2018). Cancer facts & Figures.

[2] Torre LA, Bray F, Siegel RL, Ferlay J, Lortet-Tieulent J, Jemal A (2015). Global cancer statistics, 2012. CA Cancer J Clin.

[3] Llovet JM, Bruix J (2003). Systematic review of randomized trials for unresectable hepatocellular carcinoma: Chemoembolization improves survival. Hepatology.

[4] Choi JY, Lee JM, Sirlin CB (2014). CT and MR imaging diagnosis and staging of hepatocellular carcinoma: part I. Development, growth, and spread: key pathologic and imaging aspects. Radiology.

[5] Abdelsalam ME, Murthy R, Avritscher R, Mahvash A, Wallace MJ, Kaseb AO (2016). Minimally invasive image-guided therapies for hepatocellular carcinoma. J Hepatocell Carcinoma.

[6] Lencioni R, Crocetti L (2012). Local-regional treatment of hepatocellular carcinoma. Radiology.

[7] Hinshaw JL, Lubner MG, Ziemlewicz TJ, Lee FT, Brace CL (2014). Percutaneous tumor ablation tools: microwave, radiofrequency, or cryoablation--what should you use and why?. Radiographics.

[8] Lü MD, Kuang M, Liang LJ, Xie XY, Peng BG, Liu GJ (2006). [Surgical resection versus percutaneous thermal ablation for early-stage hepatocellular carcinoma: a randomized clinical trial]. Zhonghua Yi Xue Za Zhi.

[B09] Akahane M, Koga H, Kato N, Yamada H, Uozumi K, Tateishi R (2005). Complications of percutaneous radiofrequency ablation for hepato-cellular carcinoma: imaging spectrum and management. Radiographics.

[B10] Rhim H, Yoon KH, Lee JM, Cho Y, Cho JS, Kim SH (2003). Major complications after radio-frequency thermal ablation of hepatic tumors: spectrum of imaging findings. Radiographics.

[11] Livraghi T, Solbiati L, Meloni MF, Gazelle GS, Halpern EF, Goldberg SN (2003). Treatment of focal liver tumors with percutaneous radio-frequency ablation: complications encountered in a multicenter study. Radiology.

[12] Kim SH, Lim HK, Choi D, Lee WJ, Kim SH, Kim MJ (2004). Changes in bile ducts after radiofrequency ablation of hepatocellular carcinoma: frequency and clinical significance. AJR Am J Roentgenol.

[B13] Lubner MG, Stabo N, Abel EJ, Del Rio AM, Pickhardt PJ (2016). CT Textural Analysis of Large Primary Renal Cell Carcinomas: Pretreatment Tumor Heterogeneity Correlates With Histologic Findings and Clinical Outcomes. AJR Am J Roentgenol.

[B14] Sidhu HS, Benigno S, Ganeshan B, Dikaios N, Johnston EW, Allen C (2017). “Textural analysis of multiparametric MRI detects transition zone prostate cancer”. Eur Radiol.

[B15] Vargas HA, Veeraraghavan H, Micco M, Nougaret S, Lakhman Y, Meier AA (2017). A novel representation of inter-site tumour heterogeneity from pre-treatment computed tomography textures classifies ovarian cancers by clinical outcome. Eur Radiol.

[B16] Lakhman Y, Veeraraghavan H, Chaim J, Feier D, Goldman DA, Moskowitz CS (2017). Differentiation of Uterine Leiomyosarcoma from Atypical Leiomyoma: Diagnostic Accuracy of Qualitative MR Imaging Features and Feasibility of Texture Analysis. Eur Radiol.

[B17] Ueno Y, Forghani B, Forghani R, Dohan A, Zeng XZ, Chamming's F (2017). Endometrial Carcinoma: MR Imaging-based Texture Model for Preoperative Risk Stratification-A Preliminary Analysis. Radiology.

[B18] Corino VDA, Montin E, Messina A, Casali PG, Gronchi A, Marchiano A (2018). Radiomic analysis of soft tissues sarcomas can distinguish intermediate from high-grade lesions. J Magn Reson Imaging.

[B19] Zhang Y, Oikonomou A, Wong A, Haider MA, Khalvati F (2017). Radiomics-based Prognosis Analysis for Non-Small Cell Lung Cancer. Sci Rep.

[B20] Gatenby RA, Grove O, Gillies RJ (2013). Quantitative imaging in cancer evolution and ecology. Radiology.

[21] Horvat N, Veeraraghavan H, Pelossof RA, Fernandes MC, Arora A, Khan M (2019). Radiogenomics of rectal adenocarcinoma in the era of precision medicine: A pilot study of associations between qualitative and quantitative MRI imaging features and genetic mutations. Eur J Radiol.

[22] Miranda Magalhaes Santos JM, Clemente Oliveira B, Araujo-Filho JAB, Assuncao-Jr AN, de M Machado FA, Carlos Tavares Rocha C (2020). State-of-the-art in radiomics of hepatocellular carcinoma: a review of basic principles, applications, and limitations. Abdom Radiol (NY).

[23] Haralick RM (1979). Statistical and Structural Approaches to Texture. Proceedings of the Ieee.

[24] Löfstedt T, Brynolfsson P, Asklund T, Nyholm T, Garpebring A (2019). Gray-level invariant Haralick texture features. PLoS One.

[25] Shan QY, Hu HT, Feng ST, Peng ZP, Chen SL, Zhou Q (2019). CT-based peritumoral radiomics signatures to predict early recurrence in hepatocellular carcinoma after curative tumor resection or ablation. Cancer Imaging.

[B26] Hui TCH, Chuah TK, Low HM, Tan CH (2018). Predicting early recurrence of hepatocellular carcinoma with texture analysis of preoperative MRI: a radiomics study. Clin Radiol.

[B27] Zhou Y, He L, Huang Y, Chen S, Wu P, Ye W (2017). CT-based radiomics signature: a potential biomarker for preoperative prediction of early recurrence in hepatocellular carcinoma. Abdom Radiol (NY).

[B28] Zheng BH, Liu LZ, Zhang ZZ, Shi JY, Dong LQ, Tian LY (2018). Radiomics score: a potential prognostic imaging feature for postoperative survival of solitary HCC patients. BMC Cancer.

[29] Kim S, Shin J, Kim DY, Choi GH, Kim MJ, Choi JY (2019). Radiomics on Gadoxetic Acid-Enhanced Magnetic Resonance Imaging for Prediction of Postoperative Early and Late Recurrence of Single Hepatocellular Carcinoma. Clin Cancer Res.

[30] Kim J, Choi SJ, Lee SH, Lee HY, Park H (2018). Predicting Survival Using Pretreatment CT for Patients With Hepatocellular Carcinoma Treated With Transarterial Chemoembolization: Comparison of Models Using Radiomics. AJR Am J Roentgenol.

[31] Park HJ, Kim JH, Choi SY, Lee ES, Park SJ, Byun JY (2017). Prediction of Therapeutic Response of Hepatocellular Carcinoma to Transcatheter Arterial Chemoembolization Based on Pretherapeutic Dynamic CT and Textural Findings. AJR Am J Roentgenol.

[32] Llovet JM, Zucman-Rossi J, Pikarsky E, Sangro B, Schwartz M, Sherman M (2016). Hepatocellular carcinoma. Nat Rev Dis Primers.

[33] Forner A, Reig M, Bruix J (2018). Hepatocellular carcinoma. Lancet.

[34] Kudo M, Tochio H (2008). Intranodular blood supply correlates well with biological malignancy grade determined by tumor growth rate in pathologically proven hepatocellular carcinoma. Oncology.

